# Quantifying heterogeneity in SARS-CoV-2 transmission during the lockdown in India

**DOI:** 10.1016/j.epidem.2021.100477

**Published:** 2021-09

**Authors:** Nimalan Arinaminpathy, Jishnu Das, Tyler H. McCormick, Partha Mukhopadhyay, Neelanjan Sircar

**Affiliations:** a MRC Centre for Global Infectious Disease Analysis, Imperial College, United Kingdom; b McCourt School of Public Policy and the Walsh School of Foreign Service, Georgetown University, United States; c Departments of Statistics and Sociology, University of Washington, United States; d Centre for Policy Research, New Delhi, India; e Ashoka University, Sonipat, India

**Keywords:** COVID-19, SARS-CoV-2, Heterogeneity, Transmission dynamics

## Abstract

The novel SARS-CoV-2 virus, as it manifested in India in April 2020, showed marked heterogeneity in its transmission. Here, we used data collected from contact tracing during the lockdown in response to the first wave of COVID-19 in Punjab, a major state in India, to quantify this heterogeneity, and to examine implications for transmission dynamics. We found evidence of heterogeneity acting at multiple levels: in the number of potentially infectious contacts per index case, and in the per-contact risk of infection. Incorporating these findings in simple mathematical models of disease transmission reveals that these heterogeneities act in combination to strongly influence transmission dynamics. Standard approaches, such as representing heterogeneity through secondary case distributions, could be biased by neglecting these underlying interactions between heterogeneities. We discuss implications for policy, and for more efficient contact tracing in resource-constrained settings such as India. Our results highlight how contact tracing, an important public health measure, can also provide important insights into epidemic spread and control.

## Highlights

• India’s first wave of COVID-19 in 2020 showed wide variation in the number of secondary infections per index case.
• This variation arises from variation both in the number of contacts per index case, and the risk of infection per contact.
• Transmission models capturing these factors separately can yield different dynamics to conventional approaches.
• Understanding these variations can help design more efficient approaches for contact tracing, for use in outbreak response.

## 1 Introduction

There is increasing recognition of pronounced heterogeneity in the transmission of SARS-CoV-2: that is, that the majority of transmission events appear to be caused only by a small proportion of infected individuals (Endo et al., 2020; Wang and Teunis, 2020; Gomes et al., 2020; Laxminarayan et al., 2020; Adam et al., 2020). Recent analysis has illustrated how an understanding of the drivers of this heterogeneity could have important implications for control (Sneppen et al., 2020; Liu et al., 2020). In general, heterogeneity in SARS-CoV-2 transmission is characterised through overdispersion in the secondary case distribution (Endo et al., 2020; Althouse et al., 2020; Hébert-Dufresne et al., 2020): modelling has demonstrated the important variation in epidemiological outcomes, that could arise from different levels of overdispersion in this distribution (Hébert-Dufresne et al., 2020; Lloyd-Smith et al., 2005). However, the underlying drivers for this overdispersion are not yet well-characterised. Some proposed factors include between-individual variation in infectivity (e.g. some cases may expel more virus and for longer than others), and variation in connectedness (i.e. some individuals have more contacts than others) (Althouse et al., 2020).

Contact tracing, widely implemented as part of the global response to COVID-19, including in low- and middle-income settings, can offer valuable data for examining these factors underlying the secondary case distribution. In the present work, we take advantage of contact tracing data from Punjab, a major state in India, to examine the relative roles of infectivity and connectedness, in yielding overdispersion in the secondary case distribution. The data we use here comes from the first wave of COVID-19 in Punjab in 2020, and constitutes the census of all infected persons and their contacts in the state. It was collected during a period of lockdown, thus affording a unique opportunity to measure contacts with greater accuracy than would be possible during normal economic activity. While there are inherent limits to what can be inferred from these programmatic data, our analysis shows how contact tracing data from other settings could similarly be examined. As a secondary objective, we also show how such analysis could be applied, to improve the efficiency of contact tracing: an especially pressing need in resource-limited settings. Overall, with contact tracing playing an increasingly important role in the response against SRS-CoV-2, our analysis shows how data routinely collected from these activities could be analysed, to yield insights that are important for public health, especially in resource-constrained settings.

## 2 Epidemiological context

**Fig. 1:**
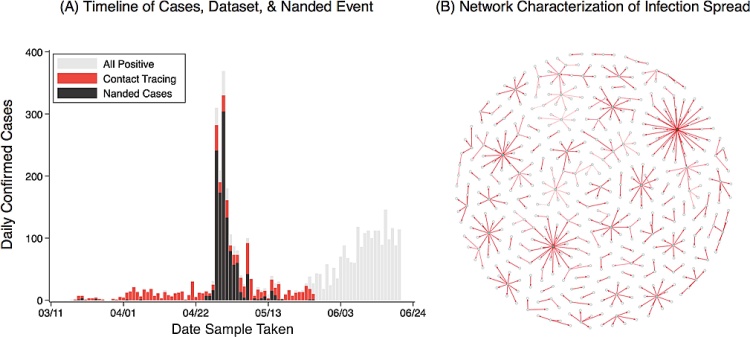
**The data from Punjab**. (A) Timeseries of reported cases in Punjab during the period of lockdown in the state (red bars) and those due to the Nanded event (black bars), and total cases from early March to the middle of June. Grey bars show additional cases that were not included in this dataset. (B) Visualisation of case clusters in the dataset (excluding the Nanded-related cases illustrated in Fig. 1A), and their linkages from self-reported contacts. Circles in black show the 454 ‘seeds’ (i.e. cases that were not known contacts of previous cases), while circles in white show cases amongst the contacts of the seeds; red lines show contact relationships. This network-type graph requires assumptions (see supporting information). Most individuals infected only few others, while a few infected many: overall, 10 % of cases accounted for 80 % of infection events (For interpretation of the references to colour in this figure legend, the reader is referred to the web version of this article).

Punjab, a state in India of about 30 million inhabitants, first went into lockdown from 1 st April to May 26th (Fig.1A) 2020. As elsewhere in India, the lockdown heavily restricted the movement of populations, in most cases to their homes and immediate neighbourhoods. Travelling outside the house required a special pass, except for essential activities which were also restricted to certain times of the day. The Government of Punjab conducted intensive contact tracing during this time, amongst all known contacts of positive cases, and regardless of symptom status. Due to the ease of tracking individuals during the lockdown, 95 % of high-risk contacts (defined as those having face-to-face conversation for at least 15 min) could be effectively traced and tested. Overall, this data constitutes the census of all infected persons and their contacts in the state between March 18, 2020 and May 26, 2020; owing to the lockdown conditions, it affords a unique opportunity to measure contacts with greater accuracy than would be possible during normal economic activity.

The data include 454 initial cases and 11,309 high risk contacts (Fig.1B). Confirmed cases comprise two groups: those residing in Punjab and who were likely infected within the state, and those who are thought to have acquired infection outside the state, due to travel or migration. Our analysis focuses on the former group, and in particular on *seeds* (the first infection in a cluster) in this group, these being the individuals amongst whom contacts are most clearly defined (see Supporting Information). This yields a total of 148 seeds with 2763 contacts; Fig. 1B illustrates the 148 seeds in black, along with the cases amongst contacts shown in white. We also present sensitivity analysis when analysing all 454 seeds with at least one contact (and all 11,309 contacts) in this data, a significant proportion (36 %) of whom were religious pilgrims who returned to Punjab from Nanded, Maharashtra, after being stranded there for a month. Notably, the vast majority (roughly 90 %) of cases in this dataset did not report symptoms. Although substantially lower than the proportion symptomatic in other settings including Western Europe (Lavezzo et al., 2020), this finding is consistent with other evidence in the India context during this period of the epidemic (Murhekar et al., 2021).

## 3 Heterogeneity in transmission

**Fig. 2:**
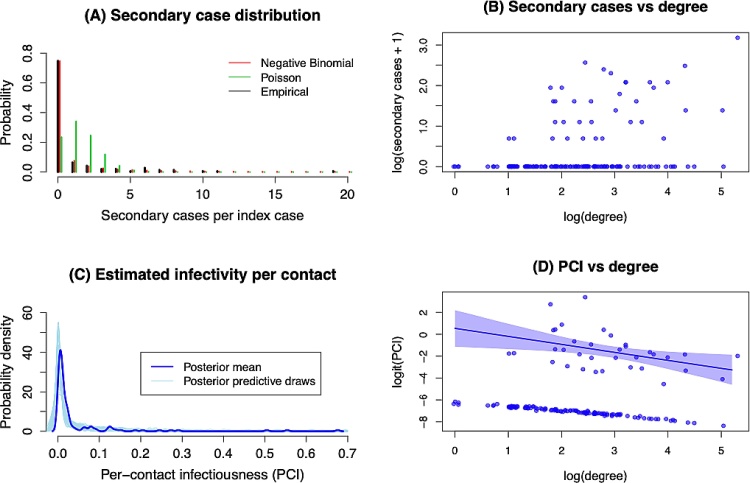
**Heterogeneity of the data in secondary cases, and in numbers of contacts**. (A) The distribution of secondary cases amongst `seeds' (i.e. first cases in each cluster shown in Fig.1B). Also shown, for comparison, are the best-fitting Poisson distribution (with), and the best-fitting negative binomial distribution (with distribution parameters). The difference between the latter two curves illustrates the strong extra-Poisson variation in the secondary case distribution. (B) Scatter plot of secondary cases vs degree, at the individual level. Shown are natural logarithms for both secondary case and degree distributions, adjusted by 1 to account for zeros, to address skewness of the distributions. Although both secondary case and degree distributions show a strong right-skew (panel A), this figure illustrates that the latter does not explain the former: despite a positive relationship between the two distributions, a substantial number of individuals with low degree generate some infections, while many with high degree generate zero onward infections. (C) Estimated marginal density of per-contact-infectiousness (PCI) that, alongside degree, is needed to explain the heterogeneity in secondary cases. Shaded intervals show 95 % Bayesian credibility intervals. (D) Estimated PCI vs degree. The figure displays relationship between the natural logarithm of the odds (logit) of PCI and the natural logarithm of the degree. These transformations allow us to plausibly model the joint distribution of PCI and degree as a multivariate normal in section 4 (see supporting information). With the empirical correlation estimated as -0.32, we adopt a range of possible values in the subsequent modelling analysis, from -0.4 to 0. There is a discernible lower band due to a large number of cases with zero onward infections, which have very low estimated PCI. Among those with onward infections, there is a discernible negative association.

The ‘’secondary case distribution” is the distribution for the number of onward infections caused by an infected individual. We observe both the number of secondary cases for each individual, and the total number of contacts the person has. In mathematical modelling of transmission dynamics, heterogeneity in transmission is conventionally captured through modelling the secondary case distribution with a negative binomial distribution, allowing for extra-Poisson variation (Endo et al., 2020; Lloyd-Smith et al., 2005). Fig. 2A illustrates the secondary case distribution in the data from Punjab. An important feature in this distribution, consistent with earlier findings (Laxminarayan et al., 2020), is that the majority (76 %) of infected cases shows no evidence of onward transmission amongst any of their contacts. A negative binomial distribution with parameters R = 0.067 and k = 0.1 captures these individuals, as well as the right-hand tail of the distribution, for example the 10 % of individuals accounting for about 80 % of transmission in this data. However, we show that this distribution conceals further levels of heterogeneity, that can be important for epidemiological outcomes.

Heterogeneity in transmission can arise from both biological and behavioural factors, including connectedness (the individuals with the most contacts having the most opportunities for transmission), and individual-level variation in infectiousness (for example, with between-individual and temporal variation in viral shedding (Fu et al., 2020; Qi et al., 2020). Fig. S2 (supporting information) illustrates the distribution in the number of reported contacts per infected case (the `degree distribution') in our dataset, showing a pronounced right-skew similar to that of the secondary case distribution. However, this skew alone cannot explain the heterogeneity in the secondary case distribution: Fig.2B shows that there are many individuals in this data set who caused no further infections despite having many contacts (i.e. having `high degree'), and conversely many individuals with low- and moderate-degree who caused several onward infections. These data suggest that there is further heterogeneity acting at the individual level, modifying the effect of the degree distribution (see also Fig. S3).

To capture this heterogeneity we defined the ‘per-contact infectiousness' (PCI) as the probability that a given contact results in infection, a probability assumed to vary by index case, but to apply equally to all contacts of a given index case. We also defined ‘zero-infectors’ as those cases with no evidence of onward infection. As shown in Fig.2B, there are several individuals with 1–2 contacts who caused zero onward infections, giving rise to substantial uncertainty in their true PCI (similar challenges apply to low-degree individuals who infected all their contacts). To address this issue we treated PCI as an individual-level effect and estimated it using Bayesian shrinkage, a technique employed (among other places) in the education statistics literature to estimate teacher effectiveness (Mendro, 1998; Lockwood et al., 2007a,b). Fig.2C shows resulting estimates for the marginal distribution of PCI over the population, once again illustrating a strong right-skew. Fig.2D illustrates this association between degree and PCI, showing: (i) a bimodal relationship between the two, arising from the large proportion of individuals that do not infect any others, and (ii) amongst those that do infect others, a negative association between degree and PCI. Estimating the gradient of the solid blue line shown in Fig. 2D (assuming independence between this gradient and zero-infectors) yields an estimate of -0.71 [-0.18, -1.24]. Estimating the correlation between log-degree and logit-PCI gives a correlation coefficient of -0.32.

The figure also illustrates a negative association even amongst zero-infectors: this association arises from the fact that, amongst zero-infectors, high-degree individuals have stronger statistical support for low PCI, than low-degree individuals. On the other hand, the negative association between PCI and degree for those who do infect others is a feature that is independent of the Bayesian shrinkage: Figure S4 in the appendix illustrates that this negative association remains when comparing the ranks of both ‘raw PCI’ (i.e. taking a simple ratio of secondary cases and degree, without Bayesian shrinkage) and degree.

Overall, these findings illustrate that overdispersion in the secondary case distribution cannot be explained by either the degree distribution or PCI alone, but rather though their joint effect. Performing these analyses on the full data for seeds (including returnees as well as the `core' group) shows qualitatively similar results (see Fig. S5).

## 4 Implications for transmission dynamics

We asked: how do outbreak dynamics compare when taking the conventional approach of using the secondary case distribution alone (Fig. 2A) vs when modelling both PCI and degree separately (Fig. 2D)? We modelled the log-transformed degree and logit-transformed PCI as following a bivariate normal distribution, with correlation ρ (see Table 1, and supporting information). Consistent with the empirical correlation of -0.32 in Fig.2D, we assumed a range of values for ρ, from -0.4 to 0. Further, as an example of a model capturing only the secondary case distribution (and not the underlying roles of degree and PCI), we adopted a negative binomial distribution for the secondary case distribution, with parameters R = 0.067, k = 0.1, consistent with that shown in Fig. 2A. Fig.S5 in the supporting information shows comparisons between these different models and the available data, for: (i) the proportion of cases that cause zero onward infections, and (ii) the empirical secondary case distribution. As illustrated by the figure, the negative binomial model shows reasonable agreement with both elements of the data. Amongst the multivariate normal models (capturing the separate roles of degree and PCI), a correlation of agrees most closely with the proportion zero-infectors.

**Table 1:** **List of the different models used, for capturing heterogeneity in the population.** 'Secondary case distributions' (models 1 - 2) are as in Fig. 2A. They ignore any interactions between degree and PCI, and instead aim only to capture variation in the numbers of secondary cases per index case. By contrast, ‘Joint distributions’ aim to model the associations shown in Fig. 2D. They employ the bivariate normal distribution described in the supporting information, with correlation ρ.

**Model number**	**Description**
**1**	Secondary case distribution using Negative Binomial distribution with number of successes = 0.1 and probability of success = 0.067
**2**	Joint degree/PCI distribution with ρ=-0.4
**3**	Joint degree/PCI distribution with ρ=-0.2
**4**	Joint degree/PCI distribution with ρ=0

**Fig. 3:**
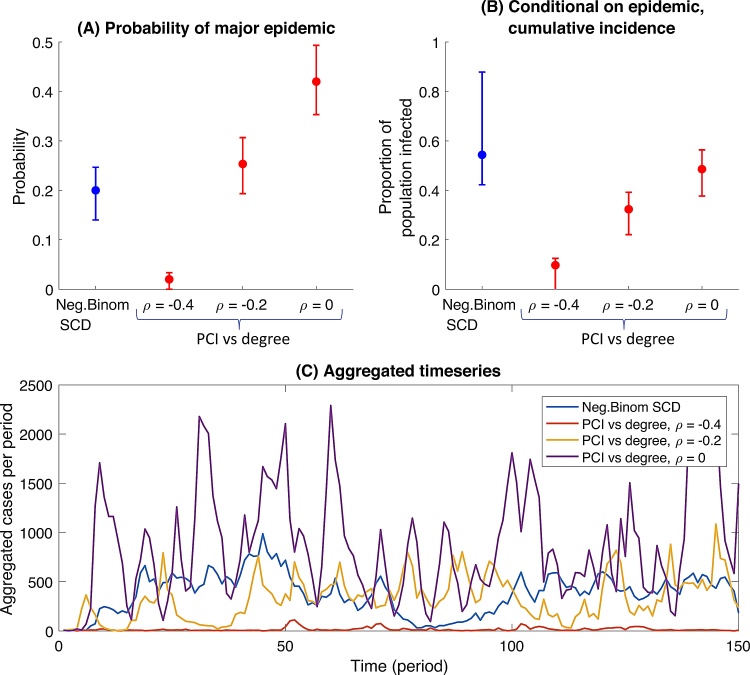
**Results of simple transmission models incorporating heterogeneity.** Model numbers are as listed in Table 1. (A, B) Epidemic outcomes over 500 time periods, assuming a 1% probability per time period, of exogenous introduction of an infectious case (here, an `epidemic' is denoted as any simulation having a cumulative incidence > 500 cases (see supporting information for rationale)). Uncertainty intervals arise from repeating simulations 250 times, and reflect 95 % simulation intervals. (C) Modelled timecourse of incidence, when aggregated over 250 simulations (with each simulation being interpreted here as an independent location).

To examine the implications of these different distributions for epidemic dynamics, we implemented a simple network simulation, in an assumed population of 3000 individuals, consistent with the population size in this study. For simplicity and generality, we simulated the epidemic in generations of infection (see supporting information). Figs. 3A,B illustrate notable variation in epidemiological outcomes, depending on whether simulations use the secondary case distribution alone (in blue) vs capturing the degree and PCIs separately (in red). In particular, zero correlation between degree and PCI yields a higher probability of a major epidemic than when using the secondary case distribution alone, and conversely for (Fig.3A). Similarly for the cumulative incidence conditional on an epidemic, the case ρ=-0.4 yields consistently smaller epidemics than when using the secondary case distribution alone, while the case ρ=0 yields comparable epidemic sizes (Fig.3B).

Fig. 3C shows additional comparisons in terms of the aggregate temporal pattern that models predict, when aggregated over multiple independent locations. Increasingly positive correlation between degree and PCI yields outbreaks with increasingly sizeable outbreaks; overall, these results highlight the wide variation in epidemiological outcomes that could arise when capturing the separate roles of degree and PCI, compared to modelling at the level of the secondary case distribution. Below we discuss reasons for this variation in behaviour.

## 5 Efficiency of contact tracing

**Fig. 4:**
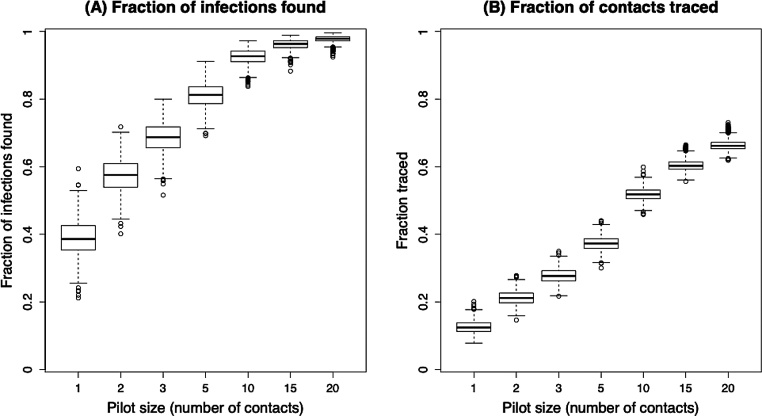
**An approach to efficient contact tracing**. Figure shows simulated outcomes of a strategy to test all contacts of an index case, only if there is at least one positive individual in an initial `pilot' sample of *s* contacts. (A) The proportion of infections found as a function of *s* (B) Overall contact tracing effort, as measured by the proportion of contacts that would be traced, again as a function of. Owing to the right-skew of the PCI, the left-hand panel illustrates diminishing returns with increasing *s*, suggesting, for example, that it would be possible to identify 80 % of the cases in this dataset, with <40 % of the contact tracing effort.

Although contact tracing plays an important role in the SARS-CoV-2 response, in resource-constrained settings such as India, its demands on the healthcare system can make it difficult to sustain. Motivated by our findings, we propose reframing contact tracing with the goal of efficiently identifying individuals with high PCI. In our data overall, we estimate that if an individual caused at least one onward infection, there is a 79 % chance that they caused at least two onward infections. We thus propose a sequential strategy where, for every index case, a `pilot' subset of only *s* randomly selected contacts are first tested; the remainder of contacts are then followed up and tested, only if there is a positive in the pilot subset. Such a strategy could substantially reduce the overall contact tracing effort, while still effectively identifying high PCI individuals. Fig. 4 shows results of simulating such a strategy 1000 times on the full dataset of 454 cases, for a range of values of *s*. The figure illustrates diminishing returns in the fraction of infections found, beyond a pilot subset size of 10 contacts (Fig. 4A). However, compared with a comprehensive strategy in which all close contacts are tested (the approach that generated this data), an alternative strategy with a pilot subset size of only 5 contacts can identify 80 % of infections (Fig. 4A), with <40 % of the contact tracing effort (Fig. 4B).

## 6 Discussion

We have shown how individual-level data, gathered from the routine course of contact tracing, can be analysed to gain important insights into the transmission of SARS-CoV-2. As well as affirming findings from elsewhere, that the majority of cases appear not to infect any others (Endo et al., 2020; Laxminarayan et al., 2020), our findings also highlight how heterogeneity in transmission may be more complex than previously recognised. This data was collected during the first wave of COVID-19 in Punjab in 2020, and we caution that our findings may not necessarily generalise to India’s ongoing ‘second wave’.

Nonetheless, simple dynamical models highlight the important role that is played by these heterogeneities. The negative binomial distribution, conventionally used in the modelling literature, captures well the proportion of individuals who do not infect others (Fig. 2A). However, our analysis also highlight some limitations of this distribution: accounting for underlying correlations between degree and PCI can lead to different outbreak dynamics, in terms of the risk and size of major outbreaks over time (Figs. 3A,B). Indeed, these results highlight the key role played by the correlation between degree and PCI, in epidemiological outcomes: when this correlation is strongly negative (ρ=-0.4), the most well-connected individuals have a concomitantly low risk of infection per contact, mitigating their role in spreading infection. Conversely, increasing ρ to 0 allows greater opportunities for the most well-connected individuals also to be the most infectious, thus giving rise to more outbreak-prone populations (Figs. 3A,B). Modelling at the level of the secondary case distribution alone cannot capture these nuances, that are consequential for modelled dynamics; in future, our results suggest that larger datasets, allowing more refined estimates of the correlations observed here, could be invaluable in allowing more accurate modelling of transmission heterogeneities, than relying on the secondary case distribution alone.

Our results also have implications for the efficiency of contact tracing. When a large fraction of infected individuals do not cause onward transmission, we show the value of a simple two-step strategy, of, for example, first testing family members and then testing other contacts only if at least one family member is found to be positive (Fig. 4). Such approaches can be particularly valuable in resource-constrained settings such as India, in decreasing the requirements for contact tracing substantially, while still identifying most cases. Fig.S7 in the supporting information shows an alternative representation of these results, plotting the relative contact tracing effort vs relative yield of secondary cases detected.

An important question, that we are not able to address using the current data, is what drives the heterogeneity in per-contact infectiousness. This heterogeneity may arise, for example, from biological factors such as the role of pre-existing, cross-reactive immunity that may moderate viral load in some individuals more effectively than others (Le Bert et al., 2020). Our analysis suggests that PCI increases with age and is significantly associated with sex (Fig. S8), although the available data does not permit a mechanistic explanation for these associations. Further data on these and other individual-level characteristics would be invaluable in further examining key risk factors for infectiousness. Where risk factors involve individual characteristics that can be readily identified in newly diagnosed patients, such as viral load, these factors could also play an important role in guiding future contact tracing efforts.

Heterogeneity in PCI could also reflect variations in the closeness of reported contacts, with some reporting only the closest contacts and others reporting wider contacts, thus explaining the negative correlation. We emphasise that our data is limited to defined `high-risk' contacts (see supporting information), thus excluding incidental contacts that might be expected to bias our estimates the most. Nonetheless, a limitation with the data is that it does not allow us to distinguish, for example, contacts having 15-minute conversations from contacts living in the same residence as the index case. For any future, similar data having this information, our analysis offers an approach for adjusting for these variations, when interpreting what routine contact tracing data means for transmission: in this case our estimates for PCI should be regarded as a data-driven weighting of contacts, rather than infectiousness. Our approach can easily be adapted for any dataset where there is additional information on closeness of contact.

Another possible driver for the heterogeneity in PCI is variation in the timing of contacts, relative to the viral dynamics of infection. For example, recent work has highlighted that SARS-CoV-2 viral load (and thus presumably infectivity) is highest just before symptom onset (Goyal et al., 2021; Cevik et al., 2021). Individuals who have frequent contacts at around this time in the clinical course may be expected to cause the greatest number of secondary infections; conversely, high-degree individuals who make most of their contacts towards the end of their symptomatic period, when viral load has substantially decreased (for example, through attendance at a gathering) may cause only relatively few onward infections. Another possible factor relating to timing is potential variation in the timeliness with which index cases were identified and then isolated (late isolation being more likely to be associated with fewer secondary cases). However, an important feature in our data is that the majority of cases did not report symptoms, thus complicating efforts to identify the timing of exposure amongst contacts. In future, to better understand the dynamics of transmission from asymptomatic individuals during the natural history of infection, prospective household cohorts with longitudinal virological and serological testing would be invaluable.

While primary data generation can be resource-intensive and complex to implement, simulation-based approaches may also be informative in understanding the drivers of heterogeneity in PCI. In particular, such approaches could be extended to simulate each of the possible factors listed above, to create ‘modelled data’ that – when subjected to the analytical methods that we have presented here – would allow examination of the plausible contribution of each of these factors. Unlike the simple branching models used in Fig.3, such simulations would need to take full account of the natural history of SARS-CoV-2 infection.

Amongst other limitations, the contact tracing data was collected, not under controlled study conditions, but as part of a public health response, by the Government of Punjab. Our approach to this data is pragmatic, recognising some inherent limitations: there may be false negatives in the data if people were tested too early, or indeed if people had been infected long in the past, which we cannot tell in the absence of serological tests. As with any contact tracing data, our assumptions for who-infected-whom in a given contact pair may be imperfect. We are able to address some of these concerns (for instance, by showing that our results are robust to a change in the directionality of a link [see supporting information]). However, more–and better–data are absolutely necessary to refine our estimates, particularly on the nature of the correlation between degree and PCI. Further, although the lockdown conditions facilitate an in-depth analysis of transmission amongst contacts, our findings must be interpreted with caution in scenarios with uninhibited transmission, as might occur in the absence of a lockdown or other non-pharmaceutical interventions (Brauner et al., 2020; Flaxman et al., 2020). Additional limitations on the modelling are described in the supporting information.

Overall, the methods that we have outlined here should apply to any contact tracing database and our publicly available code can be directly applied to any such data that have been collected. Contact tracing forms an integral part of the response to SARS-CoV-2 around the world: while being an important public health strategy in its own right, it can also provide invaluable information about how, and to whom, infection is being spread. Systematic analysis of this data could provide important insights to inform future, smarter strategies for the control of SARS-CoV-2.

## Author contributions

Nimalan Arinaminpathy: Methodology, Software, Formal Analysis, Writing – Original draft

Jishnu Das: Conceptualization, Validation, Writing – Review & Editing

Tyler H. McCormick: Methodology, Software, Formal Analysis, Writing – Original draft

Partha Mukhopadhyay: Conceptualization, Validation, Writing – Review & Editing

Neelanjan Sircar: Methodology, Software, Formal Analysis, Writing – Original draft

## Declaration of Competing Interest

None.
