# Supplementary material for: Quantifying heterogeneity in SARS-CoV-2 transmission during the lockdown in India
Source: Epidemics. 2021 Sep;36:100477. doi: 10.1016/j.epidem.2021.100477 (PMC8219474; doi:10.1016/j.epidem.2021.100477)
Supplement: Supplementary file 1 [file mmc1.docx]

**Quantifying heterogeneity in SARS-CoV-2 transmission during the lockdown in India**

Nimalan Arinaminpathy^1^, Jishnu Das^2^, Tyler H. McCormick^3^, Partha Mukhopadhyay^4^, Neelanjan Sircar^4,5^

^1^ MRC Centre for Global Infectious Disease Analysis, Imperial College

^2^ McCourt School of Public Policy and the Walsh School of Foreign Service, Georgetown University

^3^ Departments of Statistics and Sociology, University of Washington

^4^ Centre for Policy Research, New Delhi, India

^4,5^ Ashoka University, Sonipat, India

**Supporting information**

1. **Contact tracing**

The contact tracing, implemented by the Integrated Disease Surveillance Program in the state, was conducted in the following four steps [1].

- Immediately after a confirmed case is identified, a trained epidemiologist or medical officer interviews the case and ascertains all contacts.
- Contact tracing is then completed for all contacts who have interacted with the positive case anytime in the interval between onset of symptoms minus two days, and the date of isolation, or (if unavailable) upto 14 days after symptom onset. So, if symptoms started on April 1st 2020 and the person was isolated on April 5th, all persons who were in contact with the case between March 30th and April 5th are to be traced. The data are listed with details of the contacts and this list is then shared with contact tracers for tracking.
- The epidemiologist then classifies each contact as high- or low-risk. The definition of high-risk is those who face-to-face conversations for at least 15 minutes with the positive case or physical contact. Contacts who are out-of-state are passed on to other states.
- High-risk contacts are then tested by a lab technician. Contacts who are negative and remain asymptomatic for 28 days are released from the list. For those who are positive, the listing is again initiated to trace a further generation of contacts.

| Category | Fem (%) | Symp (%) | Age Range | Age (Med) | Zero Deg (%) | Total |
| --- | --- | --- | --- | --- | --- | --- |
| Seed (Normal) | 33 | 65 | 0-84 | 40 | 5 | 184 |
| Contact (Normal | 42 | 17 | 0-91 | 35 | 64 | 470 |
| Farmer/Labourer | 20 | 9 | 8-70 | 27 | 10 | 35 |
| Returnee (Nanded) | 39 | 11 | 0-100 | 45 | 83 | 1270 |
| Returnee (Others) | 18 | 14 | 1-78 | 33 | 26 | 90 |
| Other | 26 | 29 | 1-72 | 35 | 34 | 123 |

**Table S1. Key Sample Descriptives**. We provide sample descriptives for six reasons for testing: Seed (Normal), Contact (Normal), Farmer/Labour, Migrant/Returnee (Nanded), Migrant/Returnee (Others)[those not in the Nanded event], Other. We provide information on the percentage of people in each category who are female (Fem), show symptoms (Symp), and report no high risk contacts (Zero Deg). We also display median age (Age (Med)) and the minimum to maximum age (Age Range) for each category. Missing observations are removed in this table.

In the data, we recoded the reasons for testing into six categories: Seed (Normal), Contact (Normal), Farmer/Labour, Migrant/Returnee (Non-Nanded), Nanded, Other. During the lockdown, there was a fear that those entering from elsewhere would bring the infection to Punjab. Pilgrims returning from Nanded (described in section 2), other migrants/returneees, and farmers/laborers (residing in Punjab but originally from outside it) were tested by a special protocol. The ‘‘other" category consisted of certain high-risk populations like frontline healthworkers who were tested for occupational reasons and their families. The categories "Seed (Normal)" and "Contact (Normal)" correspond to those tested due to normal protocol -- usually due to symptoms, living in a containment zone, or from the contact-tracing protocol described above -- as the first case in a cluster or the contact of a confirmed case, respectively.

Table S1 displays the frequency of each reason and the percentage reporting no high risk contacts, with distributions of gender, age and symptomatic status. As we might expect laborers and migrants tend to be younger and more male. Of particular concern is the high percentage of individuals reporting no high risk contacts in most categories (Zero Deg). This is due to a bookkeeping problem.

For contacts of a previously confirmed case, we can only retrieve the number of high-risk contacts that had yet to be tested. Therefore, if person A infected person B, and both had person C as a common contact, person C would be tested as a contact of A, but would not be followed up as a contact of B. In general, this means that we do not know the total number of contacts unless the person is a ``seed" case; the total number of contacts is required for calculation of the PCI. Furthermore, returnees from elsewhere often have no contacts listed in Punjab. Accordingly, in the main text we restrict our analyses in the text to the 148 seeds tested during normal protocol that do not have a missing of zero value in the number of high risk contacts, as contacts are required to estimate PCI. Naturally, because they have come through the normal protocol, seeds have a much higher proportion of symptomatic individuals (Symp). This is a population for whom we believe we have a robust contact distribution applicable to the population of Punjab and for whom we can reliably identify seeds and contacts.

Where such information could be ascertained, we undertook an extensive exercise of matching contacts to seeds in the entire dataset, to verify the dataset had been coded correctly. Nonetheless, we were concerned about the *case ascertainment* problem. Assume that both *A* and *B* have tested positive. *B* could have infected *A* (directly or indirectly) but we observed *A* first and coded it as a seed. Two possibilities exist: either we got it right and *A* is the seed, or we got it wrong and *B* is the seed. While we can never be sure of this answer, we can test the robustness of our claims to swapping seeds and contacts. While we cannot direct compare onward infections and degree of contacts to seeds due to the bookkeeping problem, we can test whether seeds and contacts display similar infectiousness. Indeed, we see that that the contacts (2763 individuals) of normal seeds have an aggregate test positivity of 6.0% while the contacts (1885 individuals) of normal ‘‘contacts" have a test positivity of 6.5%. These are statistically indistinguishable (*p* = 0.45). Thus, as long as the coding of seed and contact by the government of Punjab was independent of degree (which is likely because seeds were typically tested due to a biological criterion -- showing symptoms -- and not a social criterion), we surmise that our estimates of the secondary case distribution are likely to be robust to swapping seeds and contacts.

Beyond the core dataset, as table S1 shows, the majority of positive cases are from the Nanded event, from which pilgrims were brought back on dedicated buses. This group is akin to the Diamond Princess experience, where multiple people were in contact with each other in close quarters. As such, seeds are contacts were not well-defined in this population.

Nevertheless, in Fig S5 in the supporting information, we also show robustness when extending our analysis to the 454 seeds across the Nanded event and all other categories (as seeds are also present in each of the four special protocol categories) who report at least one contact.

1. **Bayesian shrinkage**

A natural estimate of PCI for person $i$, $p_{i}$ would be to divide the number of onward infections ($z_{i}$) by the number of contacts ($d_{i}$), i.e., $\hat{p_{i}}=z_{i}/d_{i}$. The shape of the degree distribution presents a challenge for this method, however, as it means the variability in the estimated PCI varies across individuals based on their number of contacts. As an example, consider two individuals, *A* and *B*, with 2 and 100 contacts, respectively who have infected no one. We are confident that *B* has a PCI close to zero but not so confident with *A* due to a small sample size. We address this issue through *Bayesian shrinkage*. In this setting, individual estimates of PCI ($p_{i}$) from high contact individuals (such as *B*) will be mostly unchanged while those from lower contact individuals (such as *A*) will be shrunken towards the overall mean [2].

Amongst different ways of performing Bayesian shrinkage (e.g., the Beta-Binomial model), we chose to model the logarithm of the odds (logit) of the PCI as following a normal distribution with a common mean and variance, as this functional form is closely linked to our modelling of transmission dynamics (other approaches yield similar estimates [3]). In particular, we estimate:

$$p_{i}=logit^{-1}\left( \alpha_{i} \right)=\frac{1}{\left( 1+e^{-\alpha_{i}} \right)}$$

$$\alpha_{i}\sim Normal\left( \bar{\alpha},\sigma_{\alpha}^{2} \right)$$

where $\alpha_{i}$ is the log-odds of $p_{i}$ and $\bar{\alpha}$ is the common mean. Above, $\sigma_{\alpha}^{2}$ is inversely correlated to the amount of shrinkage. As $\sigma_{\alpha}^{2}\to0$, each $\alpha_{i}$ is given the same value, so each $p_{i}$ is estimated as the mean infection rate. As $\sigma_{\alpha}^{2}\to\infty$, $p_{i}\approx z_{i}/d_{i}$. In practice, the ‘‘hyperparameters" like $\sigma_{\alpha}^{2}$ and $\bar{\alpha}$ are estimated using Markov Chain Monte Carlo (MCMC) methods with diffuse priors. The non-zero values of $\sigma_{\alpha}^{2}$ and $\bar{\alpha}$ guarantee that our estimated $p_{i}$ is between 0 and 1.

In future work with additional data, it may be possible to characterize the complete joint distribution of PCI and number of contacts, thus alleviating the need to shrink to a common mean. For example, healthcare workers with training in mitigating infectious disease spread may have many contacts, but lower PCI than would be expected from the rest of the population. In such a setting, shrinking towards a single mean would underestimate the heterogeneity in the PCI distribution. Most of the extreme cases are 0's in the data from Punjab, however, so in practice these values will be shrunk towards a small, non-zero value under either model.

1. **Mathematical modelling of transmission dynamics**

We implemented a simple network simulation, in an assumed population of 3,000 individuals (consistent with the population size in this study). For simplicity we modelled all networks as random, that is, neglecting clustering and other forms of network structure of higher order than the degree distribution. Also for simplicity, we simulated the epidemic in terms of generations of infection, rather than in continuous time: our projections could be interpreted as being conducted in discrete time, with a time interval corresponding to the mean generation time. The focus of this modelling analysis is to understand the importance of degree distribution and PCI for transmission dynamics in general; we thus did not model the details of symptomatic vs asymptomatic infection for SARS-CoV-2, nor of the pronounced variation of severity by age [4].

*Network construction*

For the Poisson and negative binomial secondary case distributions in Table 1, we drew 3,000 samples. We then constructed a random, directed network treating these samples as degrees, to construct a network of the secondary cases that any given individual would cause, once themselves infected (our results in Fig. 3 are qualitatively unchanged when assuming a directed network instead).

In figure S2, we show that the degree distribution in the data follows an approximately log normal distribution, and in our discussion of Bayesian shrinkage (above) we showed that the logarithm of the odd (logit) of PCI is constructed to follow a normal distribution. We note further that Fig. 2D and Fig. S5(D) show that the correlation between the log-transformed degree (*n*) and logit-transformed PCI (*p*) is plausibly negative for those that infect others. We consider a population of infected individuals. Since the log-transformed degree and logit-transformed PCI each follow a normal distribution and may be correlated, the natural choice for the joint degree/PCI distribution is to model the log-transformed degree and logit-transformed PCI as following a bivariate normal distribution:

$$\left( \begin{matrix} log\left( \frac{p}{1-p} \right) \\ log(n) \end{matrix} \right)\sim N_{2}\left( \mu,\Sigma\right)$$

where $\mu$ is a vector composed of the mean values for logit-transformed PCI and log-transformed degree, and we have, for the covariance matrix $\Sigma$:

$$\Sigma=\left[ \begin{matrix} \sigma_{lp}^{2} & p\sigma_{lp}\sigma_{ln} \\ p\sigma_{lp}\sigma_{ln} & \sigma_{ln}^{2} \end{matrix} \right]$$

where $\sigma_{lp}$ is the standard deviation for the logit-transformed PCI; $\sigma_{ln}$is the standard deviation for the log-transformed degree; and $\rho$ is the correlation between the two. This construction allows us to explore different hypothetical scenarios for the correlation, and their implications for outbreak dynamics, while maintaining the correct shapes of the marginal distributions for degree and PCI. We posed three scenarios for $\rho$, taking values of -0.4, -0.2 and 0. The model we use for simulation differs from our Bayesian shrinkage approach presented in Fig. 2D. This distinction is necessary because, in our simulations, we wish to explore variation across multiple similar contact and PCI distributions. In any data analysis, however, we will model *conditional* on a particular observed contact distribution.

In a given simulation, we then sampled 3,000 values for degree and PCI. We constructed a random, undirected network from the degree distribution. For each individual *m*, we assumed that the sampled PCI $p_{m}$ applies uniformly to all of their contacts. Thus, although the link between any two individuals A and B is undirected - representing a bidirectional transmission risk - the transmission intensity is not necessarily the same in both directions, and depends on the respective PCIs of A and B (owing to between-individual variations in infectivity).

*Epidemic simulation*

For a given population constructed as above, suppose $C_{t}$ is the set of individuals that are infective at the beginning of time-step *t*; $S_{t}$ is the set of individuals that have not yet had infection and are therefore susceptible; and $J_{t}$ is the set of individuals that are newly infected in timestep *t*. Further, suppose that $p\left( m \right)$ is the sampled PCI for individual $m$. Then we proceeded along the following iterative steps:

While $t\leq500$ and $S_{t}, C_{t}$ both have at least one member:}

1. Identify $C_{t}$ with $J_{t-1}$, and initialise $J_{t}$ as an empty set
2. For every member $m$ of $C_{t}$:
   - Determine all contacts of $m$who belong to $S_{t}$ (for Models 1,2, regarding ‘contacts' as secondary cases).
   - For each such contact, conduct a Bernoulli trial with probability $p\left( m \right)$, to determine whether infection occurs (for Models 1,2, taking $p\left( m \right)=1$).
   - Accumulate all new infections thus occurring in $J_{t}$, and remove them from $S_{t}$.
3. Perform a Bernoulli trial on all members of $S_{t}$ with probability 0.01, to identify exogenous introductions of infection. Accumulate all new infections thus occurring in $J_{t}$, and remove them from $S_{t}$.
4. Increment $t$ by 1, and iterate from (1).

We repeated this algorithm 250 times, for each of the models listed in Table 1. Figure S1 shows the frequency distributions for the cumulative incidence thus obtained, i.e. the total cases over all 500 timesteps. The figure illustrates a bimodal pattern of outbreak sizes, with the vertical dashed line (at 500 cumulative cases) illustrating a consistent dividing line between the two modes. Accordingly in Figs. 3C,D in the main text, we denote ‘major epidemics' as any simulation in which cumulative incidence exceeds 500 cases.

**Figure S1: Frequency distributions for cumulative incidence**, over 500 timesteps, for each of the models listed in Table 1 in the main text. Vertical dashed lines indicate a cumulative incidence of 500, a consistent dividing line between the two modes in these distributions. Thus Fig. 3C in the main text shows the probability mass to the left of this line, while Fig. 3D shows the mean cumulative incidence to the right of this line.

*Limitations*

For simplicity we have adopted a simple network model that models the progression of an epidemic through generations of infection. This simplicity is helpful for focusing the model-based analysis on the specific types of heterogeneity revealed by our analysis of the data. It also has the benefit of generality, showing epidemiological behaviour that would apply to any disease with the same underlying heterogeneity, regardless of the details of natural history. However, an important area for future modelling work would be to incorporate some important characteristics in the natural history of SARS-CoV-2, such as symptom status, age, and the full spectrum of severity of infection [4,5]. Such refined models would be important, for example, in translating our simulated dynamics to timescales more specific to SARS-CoV-2. As mentioned above, we also take a simplified approach to the network structure, assuming the simplest case of a random network, and thus ignoring the potential for clustering, or other types of network topology that could be influential in transmission dynamics [6]. Further data on the underlying contact structure, including the retention of information on test-negative contacts, would be helpful in addressing these simplifications.

1. **Modelling efficient contact tracing algorithms**

We used the following algorithm to perform the simulations for Fig. 4. First, we choose *s*, the number of ‘pilot' contacts to be tested. Then, for each of the cases in the data from Punjab take the number of observed cases and contacts and create a vector of 1's and 0's to designate, respectively, infected contacts and those not infected. We randomly assign the position of each of the infected cases in the vector and set the number of infected cases equal to the number observed in the data for that case. We then take *s* samples without replacement and with uniform probability from the constructed vectors. If at least one of the sampled units is positive, we simulate testing the remainder of the contacts (meaning that the number of tests equals the observed number of contacts and the number of infections found equals the observed number of infections). If none of the *s* sampled contacts are positive we do not do further sampling, meaning the number of infections identified is zero and the number of tests is *s*. We repeat this exercise across all cases in the data from Punjab 1000 times for each value of *s*.

Our simulation is illustrative, with some caveats to note. First, the procedure is not optimized for the number of contacts to test. The problem we address is similar to a bandit problem in the machine learning literature where, as more information about the PCI distribution is available through testing, the number of contacts to test is optimized to maximize the (expected) number of infections found while minimizing the number of tests. We anticipate that this sequential procedure would be challenging to implement in a public health context, particularly in a low resource setting. Instead, we opt for a simple rule that can be implemented with no additional optimization (e.g. testing family members and only testing further if at least one is positive) that can still substantially improve efficiency. We also need information about the joint distribution of PCI and contacts for a fully optimized approach, which we cannot estimate with precision in the data from Punjab. Additionally, we do not consider imperfect tests. False positives would preserve the number of infections found but make the procedure less efficient since some individuals will be tested based on false positives that would not otherwise be tested. False negatives will reduce the number of infections found, though this impact would be mitigated by the right-skew of the PCI distribution. Finally, this procedure relies on the availability of tests with relatively rapid results and the willingness of individuals to be tested. If the delay between testing and receiving results is too long, then contacts who were not tested in the pilot stage could be infecting others in the population while waiting to be tested.

1. **Additional Supplementary Figures**

**Figure S2. The Contact Distribution**. Vertical bars denote the histogram of contact distribution with a bin size of 2. The density function of the log normal distribution with $\mu=2.33$and $\sigma^{2}=1.12$ fits the empirical distribution well. Consistent with standard network structure, this distribution has a strong right skew.

**Figure S3. Inadequacy of the degree distribution to explain the secondary case distribution.** While Fig. 2B in the main text illustrates this point visually, this plot offers statistical support. Red lines show the data for the secondary case distribution, while box plots show the best-fitting projections when assuming the degree distribution illustrated in Fig. S2, and moreover that the risk-of-transmission is constant across contacts (that is, a constant PCI). Doing so yields a secondary case distribution that severely underestimates the proportion of cases that caused zero onward infections.


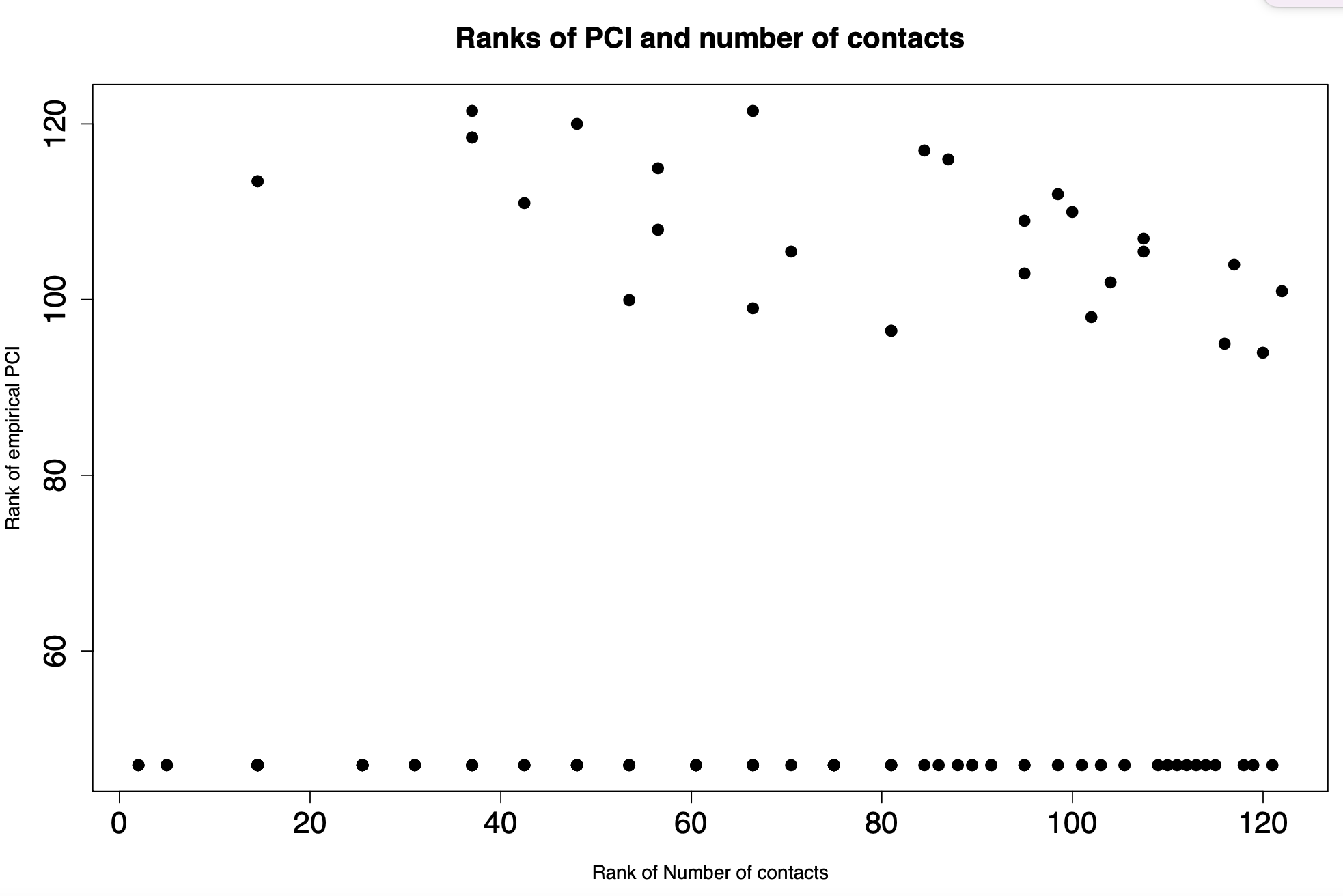


**Figure S4. Rank-rank comparison of raw PCI and degree**, where ‘raw’ PCI is calculated simply as the ratio of secondary cases to degree, without using Bayesian shrinkage. The figure illustrates a negative association between raw PCI and degree, confirming that the association shown in Fig.2D in the main text is not dependent on the use of Bayesian shrinkage to analyse this data.


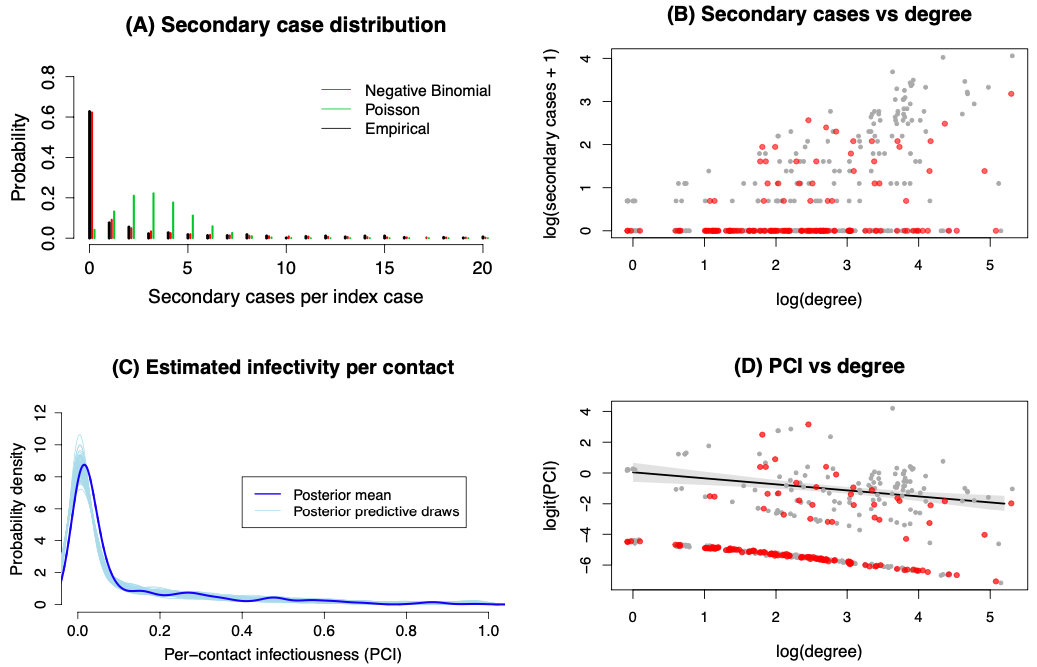


**Figure S5. Robustness for full sample**. The full heterogeneous sample is characterized in Materials and Methods. While the main text displays analysis on the core sample, the trends hold on the full dataset. Panel (A) replicates Fig. 2A for the full sample, modelling negative binomial and Poisson fits to the secondary case distribution. As before, the negative binomial fits the secondary case distribution well, while the Poisson distribution underestimates the number of zero infectors. Panel (B) shows a scatterplot of the logarithm of the degree distribution against the logarithm of the secondary case distribution adjusted by 1 to account for zeros and the skews in the distribution. The red points denote the core sample and the gray points denote the remainder of the sample. In both samples, although there is a discernible positive association between onward infections and degree, the heterogeneity in degree does not fully explain that of the secondary case distribution. Panel (C) replicates Fig. 2C for the whole sample, showing the marginal distribution of PCIs. It is right-skewed as in the core sample. Panel (D) shows the association between the log odds (logit) of the PCI and the logarithm of the degree. The red points denote the core sample and the gray points denote the remainder of the sample. Both samples are bimodal with a discernible negative association for those that infect others.

**Figure S6. Comparisons between models and data**, for the proportion of cases that cause zero onward infections (panel A) and the secondary case distribution (panel B). In both panels, dashed lines show data, and ‘Neg.Binom SCD’ denotes ‘negative binomial secondary case distribution’. For the multivariate model capturing PCI vs degree, the different values of $\rho$ correspond to the range of correlations between PCI and degree amongst individuals causing at least one onward infection, as shown in Figure 2D in the main text.

**Figure S7. Alternative representation of Figure 4 in the main text.** Here, the efficiency gains from adopting a ‘pilot driven’ approach (as described in the main text) are shown as the fraction traced vs the fraction of secondary infections found amongst contacts, both compared to a scenario where *all* contacts of an index case are followed up and tested. This figure illustrates, for example, that pilot-driven contact tracing could identify over 90% of infections amongst contacts, with approximately 55% of the effort required to test all contacts (see third point from right).

**Figure S8. Regression results for likelihood of a secondary infection, against the age, sex and symptom status of the index case**. Results are from a multivariate logistic regression. Panel (A) shows the effect of age on the probability of secondary infection per contact for the reference groups (asymptomatic, female). Probabilities are calculated as x/(1+x), where x is the age-specific odds ratio obtained from the multivariate regression. Dashed lines represent the bounds of the 95% confidence interval on the coefficients. Panel (B) shows the logistic regression coefficients (shown as log-odds ratios) for the dichotomous variables associated with being symptomatic and being male. For a person at the average age (about 42 years old) being male raises the probability of secondary infection per contact by about 0.5 percentage points (from 0.8% to 1.3%). For a person at the average age being symptomatic raises the probability of secondary infection per contact by about 1.9 percentage points (from 0.8% to about 2.7%). Recall that these estimates come from data where the majority of individuals have no secondary infections, which reduces these overall probabilities.

1. **References**

1. Indian Ministry of Health and Family Welfare. Guidance document for POEs, states and UTs for surveillance of 2019-nCoV. 2020.

2. Gelman A, Carlin JB, Stern HS, Dunson DB, Vehtari A, Rubin DB. Bayesian data analysis, third edition. Bayesian Data Analysis, Third Edition. 2013.

3. Gelman A, Hill J. Data Analysis Using Regression and Multilevel/Hierarchical Models. Data Analysis Using Regression and Multilevel/Hierarchical Models. 2006. doi:10.1017/cbo9780511790942

4. Verity R, Okell LC, Dorigatti I, Winskill P, Whittaker C, Imai N, et al. Estimates of the severity of coronavirus disease 2019: a model-based analysis. Lancet Infect Dis. 2020. doi:10.1016/s1473-3099(20)30243-7

5. Liu Y, Mao B, Liang S, Yang J-W, Lu H-W, Chai Y-H, et al. Association between age and clinical characteristics and outcomes of COVID-19. Eur Respir J. 2020;55: 2001112. doi:10.1183/13993003.01112-2020

6. Danon L, Ford AP, House T, Jewell CP, Keeling MJ, Roberts GO, et al. Networks and the epidemiology of infectious disease. Interdiscip Perspect Infect Dis. 2011/03/16. 2011;2011: 284909. doi:10.1155/2011/284909
